# PCR-Based Versus Conventional Stool Testing in Hospitalized Patients with Diarrhea: Diagnostic Yield, Clinical Impact, and Stewardship Implications

**DOI:** 10.3390/microorganisms13122785

**Published:** 2025-12-07

**Authors:** Alina Boeriu, Adina Andone, Daniela Dobru, Cristina Nicoleta Ciurea, Victoria Ancuta Nyulas, Danusia Onișor, Brindusa Tilea, Lavinia Andrada Matei, Reka-Bernadett Imreh-Ferenci, Crina Fofiu

**Affiliations:** 1 Gastroenterology Department, George Emil Palade University of Medicine, Pharmacy, Sciences and Technology of Targu Mures, 540142 Targu Mures, Romania; alina.boeriu@umfst.ro (A.B.); daniela.dobru@umfst.ro (D.D.); danusia.onisor@umfst.ro (D.O.); andrada.matei@umfst.ro (L.A.M.); crina.fofiu@umfst.ro (C.F.); 2 Gastroenterology Department, Mures County Clinical Hospital, 540103 Targu Mures, Romania; ferencibernadett1998@gmail.com; 3 Microbiology Department, George Emil Palade University of Medicine, Pharmacy, Sciences and Technology of Targu Mures, 540142 Targu Mures, Romania; cristina.ciurea@umfst.ro; 4 Department of Medical Informatics and Biostatistics, George Emil Palade University of Medicine, Pharmacy, Sciences and Technology of Targu Mures, 540142 Targu Mures, Romania; victoria.rus@umfst.ro; 5 Infectious Disease Department, George Emil Palade University of Medicine, Pharmacy, Sciences and Technology of Targu Mures, 540142 Targu Mures, Romania; brindusa.tilea@umfst.ro; 6 Internal Medicine Department, Bistrița Clinical County Emergency Hospital, 420094 Bistrita, Romania

**Keywords:** multiplex PCR, infectious diarrhea, diagnostic yield, antibiotic stewardship, hospitalized patients, mixed infections, traditional stool tests

## Abstract

Accurate and timely identification of enteric pathogens is crucial for guiding treatment in hospitalized patients with acute diarrhea. Conventional stool testing often lacks sensitivity, whereas multiplex PCR diagnostics offer rapid, comprehensive pathogen detection. This retrospective multicenter study included 267 adult inpatients with acute diarrhea from two hospitals. Patients underwent either traditional stool diagnostics (*n* = 146) or multiplex PCR testing combined with stool culture (*n* = 121). Clinical data, diagnostic yields, antibiotic use, and clinical outcomes were analyzed. The multiplex PCR group demonstrated a significantly higher diagnostic yield than traditional testing (77.7% vs. 41.1%, *p* < 0.001), detecting more mixed infections (34.7%) and a broader range of pathogens, including *Campylobacter*, viruses, and parasites. PCR positivity correlated independently with bloody diarrhea (OR 16.5; 95% CI: 1.81–150.26) and dehydration (OR 7.05; 95% CI: 1.40–35.45). PCR testing reduced inappropriate antibiotic use (OR 0.30; *p* < 0.001), shortened antibiotic duration post-result (median 5 vs. 7 days; *p* < 0.0001), and increased antibiotic adjustments (42.1% vs. 27.4%; *p* = 0.011) and discontinuation (12.4% vs. 3.7%; *p* = 0.033). The PCR group had more ICU admissions (20.7% vs. 7.1%, *p* = 0.001) and longer hospital stays (median 10 vs. 6 days, *p* < 0.0001), reflecting more severe illness. Liver cirrhosis, comorbidity burden, and systemic inflammation predicted worse outcomes. Multiplex PCR enhances pathogen detection and promotes antibiotic stewardship in hospitalized patients with diarrhea. Rapid results support earlier, targeted clinical decisions, particularly in patients with complex comorbidities or severe presentations.

## 1. Introduction

Acute gastrointestinal infections remain a significant cause of morbidity worldwide and represent a substantial burden on healthcare systems, particularly among patients with comorbid conditions or immunosuppressed states. In such populations, the rapid and accurate identification of enteric pathogens is essential to initiate appropriate antimicrobial therapy and implement effective infection control measures [1]. Conventional diagnostic methods, including stool culture, microscopy, enzyme immunoassays, and single-target PCR assays, are limited by delayed turnaround times, suboptimal sensitivity, and the inability to detect multiple pathogens simultaneously. The emergence of multiplex molecular diagnostic panels has transformed the evaluation of gastrointestinal infections by enabling rapid, sensitive, and broad-spectrum detection of bacterial, viral, and parasitic pathogens from a single stool specimen [2,3]. These assays facilitate timely clinical decision-making and enable early implementation of preventive strategies, thereby reducing the risk of nosocomial transmission and outbreaks [4]. In addition to improving diagnostic speed and sensitivity, multiplex gastrointestinal panels have been shown to enhance antimicrobial stewardship. They support more targeted antimicrobial prescribing, reduce inappropriate antibiotic use, and improve the overall efficiency of healthcare resource utilization [5]. Moreover, recent advances in molecular platforms now allow for the direct detection of antimicrobial resistance (AMR) genes from stool specimens, providing crucial data for both therapeutic management and infection control policies [6,7,8,9].

Despite these advantages, concerns remain regarding the cost-effectiveness of these molecular platforms, the clinical significance of certain detected organisms, and the potential over-reliance on molecular results [10,11].

This study aimed to compare two diagnostic strategies—traditional stool testing versus multiplex real-time PCR testing—in real-world clinical settings across two hospitals. The primary objectives were to assess differences in diagnostic yield, evaluate the impact on therapeutic decision-making, and analyze key clinical outcomes, including antibiotic stewardship, intensive care unit (ICU) admission, hospital length of stay (LOS), and in-hospital mortality.

## 2. Materials and Methods

### 2.1. Study Design and Participants

**Figure 1:**
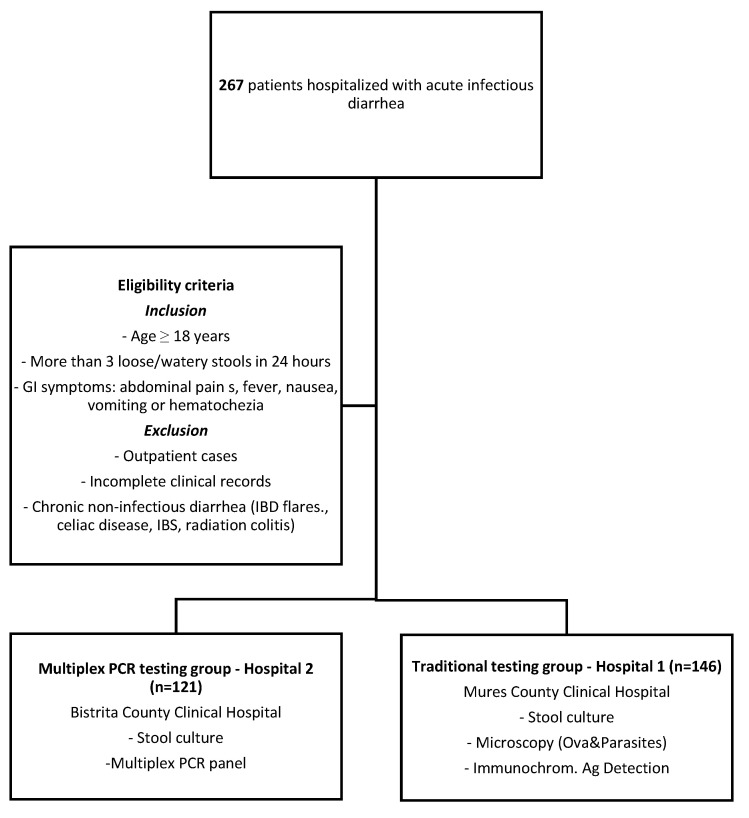
Flowchart—study design.

This retrospective, multicenter, comparative observational study included 267 hospitalized patients with suspected acute infectious diarrhea, admitted between January 2024 and January 2025. Patients were stratified into two groups based on the diagnostic approach employed. In the PCR group, 121 patients from Bistrița County Hospital (Hospital 2) underwent both conventional stool culture and multiplex PCR testing. In the traditional testing group, 146 patients from Mureș County Hospital (Hospital 1) were evaluated using standard diagnostic methods, including stool culture, microscopic examination for ova and parasites, and immunochromatographic antigen detection tests. Inclusion criteria comprised adult patients (age ≥ 18 years) presenting with ≥3 loose or watery stools within a 24 h period, accompanied by gastrointestinal symptoms such as abdominal cramping, fever, nausea, vomiting, or hematochezia. Exclusion criteria included outpatient management, incomplete clinical records, and known chronic diarrheal conditions without confirmed infectious etiology, such as inflammatory bowel disease (IBD) flares, celiac disease, irritable bowel syndrome, or radiation colitis. The study flowchart is presented in Figure 1. The study protocol was reviewed and approved by the Ethics Committees of both participating hospitals—Bistrița County Hospital (Approval No. 3690/2/25.01.2025) and Mures County Clinical Hospital (Approval No. 2721/04.03.2025).

For the purpose of this analysis, patients were categorized according to the diagnostic approach used for the stool pathogen detection: the traditional testing group (stool culture, microscopy, and antigen-based assays) and the PCR testing group (multiplex gastrointestinal PCR panels). These two diagnostic methods were compared in terms of pathogen detection rate, clinical outcomes, and antibiotic stewardship indicators.

### 2.2. Stool Analysis

#### 2.2.1. Conventional Stool Culture

Standard stool cultures were performed to identify *Salmonella*, *Shigella*, and *Yersinia enterocolitica*. Samples were inoculated on selective and differential culture media (Salmonella Shigella agar) and enriched in selenite broth. Suspected colonies were identified using biochemical tests and, when necessary, automated systems of identification (VITEK 2C, bioMérieux, Hazelwood, MO, USA). Additionally, testing was available for the same intestinal pathotypes of *Escherichia coli* (e.g., O157, O26, O55, O111, O127, O86, O119, O125, O126, O128, O25, O78, O114, O124). Sero grouping was performed with agglutination assays. For clinically significant isolates, antimicrobial susceptibility testing (AST) was conducted by the disk diffusion method and interpreted according to the EUCAST standard; Breakpoint Tables for Antimicrobial Susceptibility Testing, Version 16.0. EUCAST: Växjö, Sweden, 2025.

#### 2.2.2. Microscopy (Ova and Parasite Tests)

Microscopy (ova and parasite tests) was requested when parasitic infections were suspected. Direct wet mounts were examined under the microscope for parasite eggs, protozoan cysts, trophozoites, larvae, and adult parasites.

#### 2.2.3. Immunochromatographic Assays

Antigen-based tests (immunochromatographic assays) were available for *Clostridioides difficile*, Rotavirus, Adenovirus, *Giardia*, *Cryptosporidium*, *Campylobacter* and *Entamoeba*.

The laboratory diagnosis of *C. difficile* involved a two-step approach. Screening was performed by testing the sample for GDH (glutamate dehydrogenase). GDH-positive samples were further tested for toxins A and B.

#### 2.2.4. PCR-Based Stool Testing

Molecular analysis was performed using the DT Prime real-time PCR system (DNA-Technology, Moscow, Russia). Total nucleic acids were extracted from stool samples using the VIASURE RNA/DNA Extraction Kit (Certest Biotec, Zaragoza, Spain). Two multiplex gastrointestinal panels—Gastrointestinal Panel I and Gastrointestinal Panel II—were utilized for pathogen detection, as per manufacturer instructions. Gastrointestinal Panel I primarily targeted viral pathogens, including norovirus GI, norovirus GII, rotavirus, adenovirus, astrovirus, and sapovirus. In addition, it detected bacterial pathogens such as *Salmonella*, *Campylobacter*, *Shigella*, and *Yersinia enterocolitica*, as well as protozoal parasites like *Cryptosporidium*, *Entamoeba histolytica*, and *Giardia lamblia*. Gastrointestinal Panel II targeted key bacterial species, including *Salmonella*, *Campylobacter*, *Shigella*, *Aeromonas*, *E. coli* pathotypes (EHEC, EPEC, EIEC, and STEC), *C. difficile* (toxin B), and *Y. enterocolitica*. The panel also included parasitic organisms such as *Blastocystis hominis*, *Dientamoeba fragilis*, *E. histolytica*, and *Cryptosporidium*.

### 2.3. Data Collection Variables

The following variables were extracted from electronic medical records for all enrolled patients: demographic characteristics, comorbidities, Charlson Comorbidity Index (CCI), immunosuppression status (including underlying oncologic conditions and use of immunosuppressive therapies), recent surgical history (within the preceding 30 days), and recent antibiotic exposure (within 14 days). Clinical symptoms were recorded, including the presence of loose stools, watery or bloody diarrhea, abdominal pain, and clinical signs of dehydration. Laboratory parameters and imaging findings were assessed alongside microbiological data, which included the diagnostic method used, pathogens identified, time to result, culture positivity, and detection of *C. difficile* toxins.

Antibiotic stewardship variables encompassed empiric antibiotic use prior to test results, subsequent modification or discontinuation of therapy following microbiological confirmation, total duration of antibiotic therapy post-diagnosis, and assessment of appropriateness. In both study groups, targeted antimicrobial treatment following a positive stool result was managed under the supervision of an infectious diseases specialist, who considered the clinical presentation, immune status, comorbidities, and inflammatory biomarkers. Inappropriate empiric antibiotic use was defined as initial therapy not aligned with subsequent microbiologic findings, such as antibiotics that had to be changed with targeted antimicrobial therapy (e.g., vancomycin for *Clostridioides difficile* infection or azithromycin for *Campylobacter*) or had to be discontinued in cases of viral, parasitic, or negative results. Clinical outcome measures included length of hospital stay (LOS), admission to the intensive care unit (ICU) following stool testing, and in-hospital mortality.

### 2.4. Statistical Analysis

Statistical analyses were performed using SPSS Statistics for Windows, version 20.0 (SPSS Inc., Chicago, IL, USA). Categorical variables were analyzed using the chi-square test or Fisher’s exact test, as appropriate, to assess associations and differences between groups. The Kolmogorov–Smirnov test was applied to evaluate the normality of distribution for continuous variables. Normally distributed continuous variables were compared using the independent samples *t*-test, while non-normally distributed variables were analyzed using non-parametric methods. Categorical data were presented as absolute numbers and percentages. Continuous variables with a normal distribution were expressed as mean ± standard deviation (SD), whereas non-normally distributed data were reported as median and interquartile range (IQR). Logistic regression analysis was conducted to identify independent predictors of diagnostic test positivity, clinical outcomes, and antibiotic stewardship-related decisions. A two-tailed *p*-value ≤ 0.05 was considered statistically significant.

## 3. Results

### 3.1. Baseline Characteristics

A total of 267 hospitalized patients diagnosed with acute diarrhea were included in the analysis. Among them, 146 patients underwent traditional stool diagnostics, while 121 patients received multiplex PCR-based testing in conjunction with standard stool culture. Patients in the PCR group were significantly older than those in the traditional group (median age: 65 vs. 60 years; *p* = 0.02) and exhibited a greater burden of comorbidities, as reflected by a higher Charlson Comorbidity Index (median: 5 vs. 2; *p* < 0.0001). Comorbid conditions, including cardiovascular disease, liver cirrhosis, and renal impairment, were significantly more prevalent in the PCR group (*p* < 0.05). Likewise, the use of immunosuppressive therapy (34.7% vs. 11.5%, *p* < 0.001) and recent surgical interventions (18.2% vs. 7.7%, *p* = 0.008) were more common in patients evaluated with PCR-based diagnostics (Table 1).

### 3.2. Clinical Presentation and Laboratory Findings

Patients evaluated with multiplex PCR presented more frequently with bloody diarrhea (28.1% vs. 8.0%; *p* < 0.0001), abdominal pain (76.9% vs. 51.1%; *p* < 0.0001), and dehydration (51.2% vs. 68.6%; *p* = 0.004) compared to those assessed with traditional diagnostic methods. Inflammatory markers were significantly elevated in the PCR group, with a median C-reactive protein (CRP) level of 109 mg/L versus 4.35 mg/L in the traditional group (*p* < 0.0001). Correspondingly, hemoglobin levels were lower in the PCR cohort (11.2 g/dL vs. 12.85 g/dL; *p* < 0.0001), suggesting a more severe clinical presentation. The number of stool tests performed per patient was significantly lower in the PCR group (median 1; IQR 1–1) compared to the traditional testing group (median 2; IQR 2–3; *p* < 0.0001) (Table 2).

### 3.3. Diagnostic Yield

**Figure 2:**
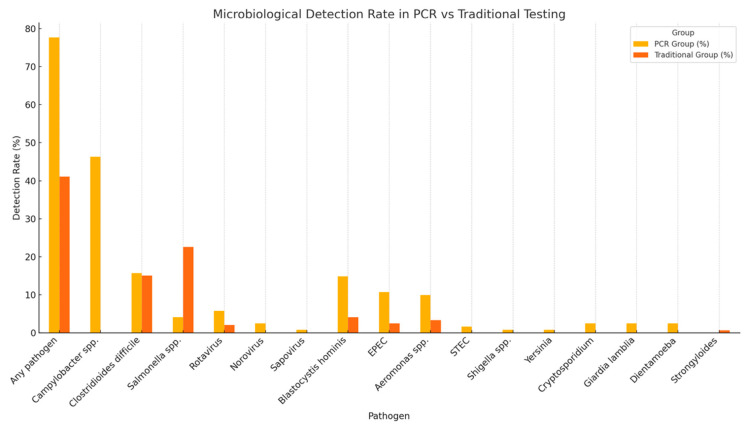
Microbiological detection rate.

**Figure 3:**
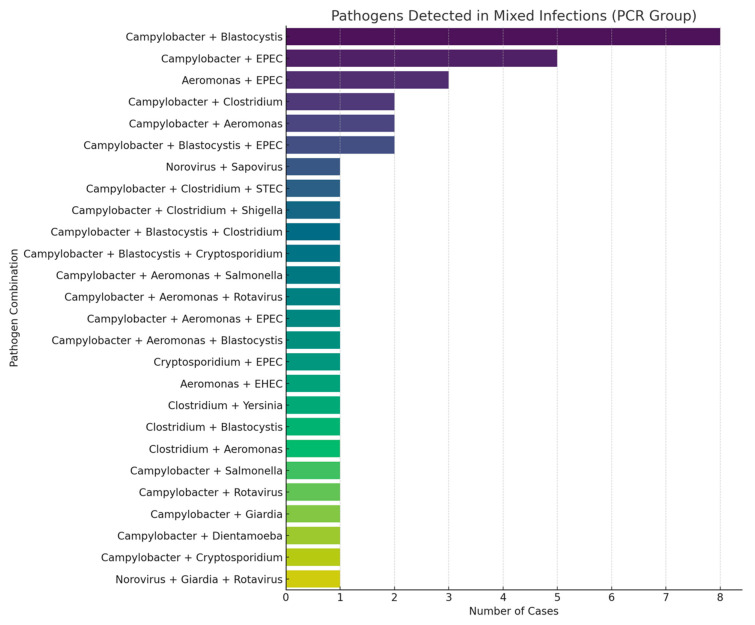
Mixed infections diagnosed by PCR stool testing.

The time to diagnostic result was significantly shorter in the PCR group, with a median turnaround time of 1 day compared to 2 days in the traditional testing group (*p* < 0.0001). Although culture positivity was higher in the traditional group (23.1% vs. 0.8%; *p* < 0.0001), the overall diagnostic yield was significantly greater in the PCR group (77.68% vs. 41.09%; *p* < 0.001) (Table 3). Multiplex PCR enabled the detection of a broader spectrum of pathogens, including bacteria, viruses, and protozoa, providing a more comprehensive diagnostic profile (Figure 2). *Campylobacter* was exclusively identified by PCR, with a prevalence of 46.28% among patients tested with this method. Additionally, mixed infections were detected in 34.7% of PCR-tested patients, including 25.6% with dual and 9.1% with triple infections. In contrast, no mixed infections were reported using traditional diagnostic methods. The most frequently observed dual-pathogen combination was *Campylobacter* with *Blastocystis hominis* (*n = *8), while the most common triple-pathogen combination included *Campylobacter*, *Blastocystis hominis*, and enteropathogenic *E. coli* (EPEC) (*n = *2) (Figure 3). *C. difficile* was detected at similar frequencies in both groups (15.7% in the PCR group vs. 15.06% in the traditional group; *p* = 0.53), indicating the reliability of both diagnostic approaches for this pathogen.

### 3.4. Predictors of Diagnostic Tests Positivity

Multivariable logistic regression analysis identified bloody diarrhea as an independent predictor of PCR test positivity (odds ratio [OR] 16.5; 95% confidence interval [CI]: 1.81–150.26; *p* = 0.013), followed by the presence of dehydration (OR 7.05; 95% CI: 1.40–35.45; *p* = 0.018). Other clinical and laboratory variables, including elevated C-reactive protein (CRP > 10 mg/L), leukocytosis, immunosuppression, and Charlson Comorbidity Index, were not significantly associated with a positive PCR result. For traditional diagnostic testing, the presence of loose stools was independently associated with higher odds of pathogen detection (OR 2.57; 95% CI: 1.01–6.55; *p* = 0.048), while watery diarrhea was inversely associated with test positivity (OR 0.27; *p* = 0.025). No other variables demonstrated statistical significance in predicting traditional test positivity (Table 4). Furthermore, no clinical or laboratory parameters were identified as significant predictors for the detection of mixed infections using PCR-based stool testing.

### 3.5. Antibiotic Stewardship Analysis

#### 3.5.1. Empiric Antibiotic Use Prior to Diagnostic Results

Empirical antibiotic administration prior to the availability of diagnostic results was significantly more common in the PCR group compared to the traditional testing group (70.0% vs. 36.3%; *p* < 0.0001). Multivariate logistic regression analysis identified several independent predictors of empiric antibiotic use. Abdominal pain was inversely associated with empiric antibiotic initiation (OR 0.47; 95% CI: 0.25–0.86; *p* = 0.015), suggesting a tendency to withhold antibiotics in patients presenting with localized gastrointestinal symptoms rather than systemic signs of infection. Likewise, patients with underlying renal disease were less likely to receive empirical antibiotics (OR 0.31; *p* = 0.01), which may reflect greater prescribing caution due to concerns about nephrotoxicity. In contrast, a higher Charlson Comorbidity Index was independently associated with increased empiric antibiotic use (OR 1.19; *p* = 0.001), as was leukocytosis > 10,000/μL (OR 2.18; *p* = 0.002), both likely reflecting a higher perceived risk of bacterial infection or severe disease. Other clinical features, including watery diarrhea, bloody diarrhea, dehydration, and ICU admission, showed no significant association with the initiation of empiric antibiotic therapy prior to the availability of test results (Table 5).

#### 3.5.2. Inappropriate Antibiotic Use

Multivariate regression analysis identified renal dysfunction as a significant negative predictor of inappropriate antibiotic use (OR 0.36; 95% CI: 0.15–0.84; *p* = 0.018). In contrast, a higher Charlson Comorbidity Index was independently associated with increased odds of inappropriate antibiotic administration (OR 1.12; 95% CI: 1.02–1.22; *p* = 0.019). The use of traditional diagnostic methods was significantly associated with an increased likelihood of inappropriate antibiotic therapy compared with multiplex PCR testing. In contrast, PCR-based diagnostics were independently protective (OR of 0.30; 95% CI: 0.16–0.53; *p* < 0.001). No statistically significant associations were observed between inappropriate antibiotic use and specific clinical symptoms, including watery diarrhea, bloody diarrhea, dehydration, ICU admission, or immunosuppression status (Table 6).

### 3.6. Impact of Diagnostic Results on Antibiotic Stewardship

Following the release of diagnostic results, PCR testing was associated with more judicious and targeted antibiotic use. Continuation of empiric antibiotic therapy after result availability occurred in 6.84% of patients in the traditional testing group, whereas no such cases were observed in the PCR group. Modification of antibiotic regimens based on test results was significantly more frequent in the PCR cohort (42.14% vs. 27.39%; *p* = 0.011), suggesting that multiplex PCR results were more likely to influence treatment adjustments based on pathogen-specific identification. Antibiotic discontinuation following diagnostic clarification was more common in the PCR group (12.39% vs. 3.70%; *p* = 0.033), highlighting the role of rapid and comprehensive diagnostics in reducing unnecessary antimicrobial exposure. The median duration of antibiotic therapy following test results was also significantly shorter in the PCR group (5 days vs. 7 days; *p* < 0.0001). Although the addition of antibiotics based on test findings occurred less frequently in the PCR group (15.7% vs. 21.23%), the difference did not reach statistical significance (*p* = 0.248), indicating comparable clinical thresholds for escalation of therapy (Table 7).

### 3.7. Clinical Outcomes and Predictors of Negative Oucomes

Patients evaluated with multiplex PCR experienced significantly longer hospital stays compared to those undergoing traditional diagnostic testing (median: 10 vs. 6 days; *p* < 0.0001). Moreover, transfer to the intensive care unit (ICU) was significantly more frequent in the PCR group (20.7% vs. 7.1%; *p* = 0.001). In contrast, in-hospital mortality rates did not differ significantly between the two groups (5.8% vs. 4.8%; *p* = 0.768), suggesting that the choice of diagnostic modality did not independently influence survival outcomes.

### 3.8. Predictors of ICU Admission

Multivariate logistic regression identified several independent predictors of ICU transfer. Liver cirrhosis emerged as the strongest risk factor (OR 6.69; 95% CI: 2.52–17.77; *p* < 0.001), followed by the presence of chronic respiratory disease (OR 3.07; 95% CI: 1.09–8.67; *p* = 0.034). Additionally, a higher Charlson Comorbidity Index (OR 1.26 per unit increase; *p* = 0.001) and longer hospital stay duration (OR 1.08 per day; *p* = 0.006) were significantly associated with increased likelihood of ICU admission (Table 8).

### 3.9. Predictors of Length of Hospital Stay (LOS)

ICU admission was the most significant predictor of prolonged hospitalization, contributing to an average increase of 5.23 days (*p* < 0.001). Elevated white blood cell count (WBC > 10,000/μL) was also associated with extended length of stay (coefficient B = 2.67; *p* = 0.004), indicating a potential link between systemic inflammation and slower clinical recovery. Each one-point increase in the Charlson Comorbidity Index was associated with an additional 0.56 days of hospitalization (*p* = 0.017). Liver cirrhosis and respiratory disease were also associated with prolonged hospital stays, though the results approached but did not reach statistical significance (Table 8). Positive stool test results were independently associated with prolonged hospitalization (OR 2.78; 95% CI: 1.16–4.39; *p* = 0.001).

## 4. Discussion

Acute diarrhea in hospitalized patients may result from infectious etiologies, adverse effects of medications, or nosocomial causes. While mild, self-limiting cases can often be managed with supportive care, the presence of clinical severity indicators, such as dehydration, abdominal pain, hematochezia, or fever, requires thorough evaluation and risk stratification. Hospitalized patients with significant comorbidities or immunocompromised states are at increased risk of complications, making timely pathogen identification and targeted therapy critical [12]. In this study, we compared the performance of multiplex PCR testing with conventional diagnostic methods in hospitalized patients with diarrhea, focusing on diagnostic yield, antibiotic stewardship, and key clinical outcomes.

Multiplex PCR achieved a significantly higher overall pathogen detection rate than conventional testing (77.7% vs. 41.1%, *p* = 0.0001) and uniquely enabled the identification of viral, protozoal, and multiple bacterial co-infections. Our findings are consistent with previous reports showing that multiplex PCR enhances sensitivity in gastrointestinal infections, improving pathogen detection, reducing time to diagnosis, and supporting targeted antimicrobial therapy [3,13,14,15]. Its ability to simultaneously identify multiple pathogens from a single specimen provides a distinct diagnostic advantage over traditional methods, which rarely detect co-infections due to limited organism coverage [14,15,16,17].

In our study, mixed infections were detected in 34.7% of patients in the PCR group, highlighting the value of multiplex testing in complex clinical scenarios. The detection of multiple enteropathogens may be attributable to prolonged pathogen shedding, particularly in immunocompromised patients [18]. However, the clinical significance of certain organisms, such as *Blastocystis*, *Dientamoeba fragilis*, and enteropathogenic *E. coli* (EPEC), remains controversial. In our study, the most common co-infection was *Campylobacter* with *Blastocystis*, a finding that supports previous observations suggesting that co-infection with bacterial, viral, fungal, or parasitic pathogens may augment the pathogenic potential of *Blastocystis* [19]. We observed no significant associations between immunosuppression or the Charlson Comorbidity Index and mixed infections, consistent with prior reports that immunosuppression does not increase the likelihood of multiple pathogen detection by multiplex stool PCR [20].

Stool culture remains a time-consuming diagnostic modality with limited efficacy in detecting slow-growing pathogens. Notably, *Campylobacter* and pathogenic *E. coli* strains are not routinely cultured in many laboratories, and rapid antigen-based assays for viral gastroenteritis are seldom performed unless specifically requested by the attending physician. In our study, diagnostic evaluation in the traditional testing group was guided by the clinician’s judgment, with investigations ordered selectively based on clinical presentation and paraclinical findings. The high proportion of negative results reflects the limited diagnostic yield of conventional stool culture for acute diarrhea, consistent with previously reported data [21].

Nevertheless, stool culture retains clinical value, particularly when it enables the identification of pathogens of public health importance and the performance of antimicrobial susceptibility testing. This is especially relevant for pathogens such as *Salmonella*, where resistance patterns can vary considerably across geographical regions [22]. Conventional culture techniques have historically identified *Salmonella* and *Campylobacter* as the most common bacterial causes of acute diarrhea [21,23]. In our study, however, only *Salmonella* infections were detected by stool culture, accounting for 33 cases (22.6%), with antimicrobial susceptibility testing performed in all positive isolates. It is noteworthy that rapid diagnostic assays were employed for the detection of *Campylobacter* infections in the conventional testing group, as the laboratory infrastructure did not support the specialized growth conditions required for *Campylobacter* culture. Multiplex PCR testing substantially enhanced the detection of *Campylobacter*, identifying 56 positive cases compared to none detected by conventional methods. This observation aligns with previous reports demonstrating the superior sensitivity of molecular diagnostics for *Campylobacter* detection in hospitalized patients [24,25]. Nonetheless, the identification of certain pathogens, particularly *Campylobacter* and enteropathogenic *E. coli* (EPEC), warrants cautious clinical interpretation. In such cases, culture confirmation following a positive PCR result is recommended to differentiate true infectious gastroenteritis from non-infectious causes of diarrhea [26]. In our cohort, *E. coli* was identified in 16 cases by PCR, with EPEC representing the most frequently detected subtype. Although EPEC infections are often asymptomatic in adults, their pathogenic potential in vulnerable populations should not be underestimated. Clinical context and adjunctive laboratory parameters, such as hyponatremia, which has been associated with EPEC infections, may help identify cases that warrant targeted antimicrobial therapy [27].

*C. difficile* emerged as the second most frequently detected pathogen in both diagnostic groups, with 22 cases identified in the traditional testing group and 19 in the PCR group. Among established risk factors for *C. difficile* infection, including recent antibiotic exposure, prior hospitalization, advanced age, and comorbidities [28,29], only recent antibiotic use was significantly associated with test positivity in both groups (OR 3.17; 95% CI: 1.37–7.37; *p* = 0.007). In accordance with current clinical guidelines, a multistep diagnostic algorithm was employed, consisting of glutamate dehydrogenase (GDH) antigen detection or PCR as an initial screening test, followed by toxin A and B enzyme immunoassays to confirm active toxin production [30,31,32]. The rapid turnaround time offered by PCR provided a distinct clinical advantage, particularly in patients with severe *C. difficile* infection, where timely therapeutic decisions are critical.

Viral and parasitic pathogens, including rotavirus, norovirus, sapovirus, *Cryptosporidium*, *Dientamoeba*, and *Giardia lamblia*, were exclusively detected by multiplex PCR testing. In contrast, traditional diagnostic methods demonstrated limited performance for viral pathogen detection, identifying only three cases of rotavirus and one case of adenovirus. These findings are consistent with previous reports highlighting the suboptimal sensitivity of conventional techniques for non-bacterial enteropathogens [33].

The paraclinical profile of patients who underwent multiplex PCR testing demonstrated a more pronounced inflammatory phenotype, characterized by elevated white blood cell (WBC) counts and C-reactive protein (CRP) levels, along with lower hemoglobin concentrations. These findings suggest that PCR testing may have been preferentially selected for patients presenting with more severe clinical features. To further evaluate predictors of stool test positivity, we conducted a multivariable logistic regression analysis. Bloody diarrhea emerged as a strong independent predictor of PCR positivity (OR 16.5; 95% CI: 1.81–150.26; *p* = 0.013), followed by clinical signs of dehydration (OR 7.05; 95% CI: 1.40–35.45; *p* = 0.018). These findings indicate that more severe gastrointestinal presentations are associated with a higher likelihood of pathogen detection by multiplex PCR, reflecting the assay’s enhanced sensitivity for invasive or inflammatory pathogens (e.g., *Campylobacter*, *C. difficile*, EPEC, or mixed infections). These observations are consistent with the findings of Kwack et al., who also reported a higher prevalence of bloody diarrhea among PCR-positive patients [25]. In contrast, within the traditional stool testing group, the presence of loose stools was the only significant clinical predictor of test positivity (OR 2.57; 95% CI: 1.01–6.55; *p* = 0.048). In this group, *Salmonella* and *C. difficile* were the predominant pathogens detected, and no cases of mixed infections were identified. Prior studies have reported that fever, prolonged or frequent diarrhea, and elevated CRP are associated with positive stool culture results [21,34]. Our comparative analysis highlights that PCR testing provides a broader and more clinically relevant pathogen detection profile, particularly in patients presenting with severe diarrhea.

We further analyzed the appropriateness, duration, and modification of antibiotic therapy in both patient groups. Empiric antibiotic use prior to the availability of diagnostic results was significantly more common in the PCR group (70.0%) than in the traditional testing group (37.1%; *p* < 0.0001). Patients in the PCR group were older, had a greater comorbidity burden, and presented with more severe illness, as evidenced by higher ICU admission rates and longer hospital stays. These factors likely contributed to the increased use of empiric antibiotics. Multivariable logistic regression further identified a higher Charlson Comorbidity Index (CCI) as an independent predictor of empiric antibiotic therapy (OR 1.19; 95% CI: 1.08–1.31; *p* = 0.001), reflecting a clinical tendency toward anticipatory prescribing in medically complex patients. Similarly, leukocytosis (WBC > 10,000/μL) was significantly associated with empiric antibiotic initiation (OR 2.18; 95% CI: 1.33–3.60; *p* = 0.002), suggesting that systemic inflammatory markers strongly influenced early treatment decisions. By contrast, classic gastrointestinal symptoms, including watery diarrhea, bloody diarrhea, and dehydration, were not significant predictors of empiric antibiotic use. This finding underscores the limited predictive value of gastrointestinal symptomatology alone and highlights the predominant role of systemic inflammation and comorbidity burden in guiding empiric antimicrobial decisions.

Inappropriate antibiotic use was observed in both diagnostic groups, with treatment modifications made following the availability of stool test results. In 15 cases where viral or parasitic pathogens were identified by multiplex PCR, empiric antibiotic therapy was appropriately discontinued. Given the rising prevalence of fluoroquinolone resistance [35] and local antimicrobial susceptibility patterns, institutional protocols recommend a five-day course of azithromycin for the treatment of *Campylobacter* infections. Accordingly, empiric therapy with cephalosporins or ciprofloxacin was deemed inappropriate in 35 cases and was subsequently modified following pathogen-specific identification. In patients with confirmed *C. difficile* infection, empiric treatment with cephalosporins or fluoroquinolones was discontinued and replaced with guideline-recommended therapy, typically oral vancomycin with or without metronidazole. Overall, antibiotic regimens were modified in 8 patients in the PCR group and 14 patients in the traditional testing group after confirmation of *C. difficile* infection.

Logistic regression analysis evaluating predictors of inappropriate antibiotic use identified only a limited number of statistically significant associations, highlighting the complexity of antimicrobial decision-making in hospitalized patients. Renal dysfunction was independently associated with a reduced likelihood of inappropriate prescribing (OR 0.36; 95% CI: 0.15–0.84; *p* = 0.018), reflecting greater prescriber caution due to concerns about nephrotoxicity and adherence to renal-specific stewardship protocols. Conversely, a higher Charlson Comorbidity Index was positively associated with inappropriate antibiotic use (OR 1.12; 95% CI: 1.02–1.22; *p* = 0.019), suggesting that increasing patient complexity may drive broader or prolonged empiric coverage. PCR-based testing was independently associated with a significantly lower likelihood of inappropriate antibiotic prescribing compared to traditional diagnostic methods (OR 0.30; 95% CI: 0.16–0.53; *p* < 0.001).

The shorter turnaround time for results in the PCR group (median 1 day vs. 2 days; *p* < 0.0001) facilitated earlier clinical decision-making and the prompt implementation of infection control interventions, such as patient isolation, thereby reducing the risk of nosocomial transmission. Integration of multiplex PCR testing into clinical workflows significantly improved stewardship outcomes by increasing diagnostic confidence. Compared with the traditional testing group, PCR-tested patients had lower rates of antibiotic additions (15.7% vs. 21.23%), higher rates of early antibiotic discontinuation when infection was excluded (12.39% vs. 3.7%; *p* = 0.033), shorter duration of antibiotic therapy after diagnostic results (mean 5 days vs. 7 days; *p* < 0.0001), and more frequent post-result antibiotic adjustments (42.14% vs. 27.39%; *p* = 0.011), reflecting more precise implementation of pathogen-directed therapy. The frequency of antibiotic additions after diagnostic results did not differ significantly between groups (15.70% vs. 21.23%; *p* = 0.248), suggesting that escalation decisions were driven primarily by pathogen identification rather than by diagnostic modality. Patients in the traditional testing group were more frequently exposed to empiric and prolonged antibiotic therapy, with fewer instances of appropriate antibiotic discontinuation. These trends likely reflect the limited diagnostic yield, longer turnaround time, and higher frequency of inconclusive or negative results associated with conventional stool testing, which collectively favored the continuation of empiric treatment.

Previous studies evaluating the impact of multiplex gastrointestinal PCR panels on antibiotic stewardship have reported mixed findings. While some investigations observed no significant differences in antibiotic duration or modification compared with traditional testing [36,37], other evidence indicates that timely pathogen identification supports targeted therapy [38,39], reduces unnecessary antibiotic exposure [40], and may decrease the risk of antimicrobial resistance and *C. difficile* infection. Additional benefits of multiplex PCR testing include a reduced need for ancillary diagnostic procedures, shorter durations of contact isolation, decreased hospital length of stay, and potential cost savings [5,23,41,42,43]. The diagnostic evaluation of acute diarrhea typically requires multiple standard laboratory investigations, with an average of three routine tests per patient sample [16,44]. In our study, however, the number of stool tests performed per patient was significantly lower in the PCR group (*p* < 0.0001), reflecting greater diagnostic efficiency.

Clinical outcomes revealed no significant difference in in-hospital mortality between the PCR and traditional testing groups. However, both the rate of intensive care unit (ICU) admissions and the length of hospital stay (LOS) were significantly higher among patients in the PCR group. Our analysis indicates that LOS was driven primarily by markers of systemic illness severity and the need for ICU-level care, rather than gastrointestinal symptomatology or the diagnostic modality. Leukocytosis (WBC > 10,000/µL), ICU admission and higher Charlson Comorbidity Index (CCI) scores were independently associated with prolonged LOS. Stool test positivity is also predictive of prolonged hospitalization (OR = 2.78; 95% CI: 1.16–4.39; *p* = 0.001), suggesting that infectious burden may substantially influence disease course and hospitalization duration. Liver cirrhosis emerged as the strongest independent predictor of ICU admission (OR = 6.69; 95% CI: 2.52–17.77; *p* < 0.001), followed by chronic respiratory disease (OR = 3.07; 95% CI: 1.09–8.67; *p* = 0.034) and higher CCI scores (OR = 1.26; 95% CI: 1.1–1.43; *p* = 0.001). These findings highlight the critical role of underlying comorbidities in driving escalation to intensive care. Timely microbiological diagnosis and prompt initiation of targeted antimicrobial therapy remain essential in vulnerable patients, particularly those with advanced liver [45] or pulmonary disease.

Multiplex PCR assays represent an important advance in the diagnostic approach to acute infectious diarrhea, allowing simultaneous detection of a broad range of bacterial, viral and parasitic agents from a single sample. This comprehensive detection profile enhances diagnostic accuracy and significantly reduces the time to pathogen identification, which is particularly valuable in hospitalized patients with severe or immunocompromised conditions. Beyond rapidity, molecular assays improve clinical decision-making by distinguishing infectious from non-infectious diarrhea, enabling timely infection control measures and targeted antibiotic therapy. Therefore, multiplex PCR not only increases diagnostic yield but also supports more rational use of antimicrobials.

While multiplex PCR testing offers clear diagnostic advantages, its implementation depends on available resources and laboratory infrastructure. In high-income settings, it is increasingly used as a routine diagnostic tool due to its speed and accuracy. In contrast, in resource-limited hospitals, conventional methods remain essential for their low cost and ability to provide antimicrobial susceptibility data.

Although this study compared two patient cohorts from different hospitals, each employing distinct diagnostic approaches, this design reflects a pragmatic, real-world scenario in which institutional testing protocols vary according to available resources and expertise. Similar multicenter retrospective comparisons have been successfully used to evaluate multiplex gastrointestinal PCR panels against traditional stool diagnostics in hospitalized patients [2,5,13,23,36]. While we acknowledge that this approach introduces potential heterogeneity, all analyses were carefully adjusted for demographic and clinical confounders, including comorbidity burden, inflammatory markers, and disease severity. Importantly, the observed associations between multiplex PCR testing and improved antibiotic stewardship outcomes remained statistically significant after adjustment, suggesting that the findings are robust and not solely attributable to differences in baseline characteristics. From our perspective, the higher severity observed in the PCR cohort underscores the clinical utility of rapid molecular testing, particularly in complex, high-risk patients in whom early pathogen identification is most impactful. Therefore, we consider our results both relevant and representative of the actual clinical benefits achievable when integrating multiplex PCR diagnostics into hospital practice. Future prospective or paired-sample studies are warranted to confirm these findings under standardized testing conditions.

This study has several limitations inherent to its retrospective design. The inclusion of two hospitals with differing diagnostic protocols introduces the possibility of institutional bias. Patient selection for PCR testing and therapeutic decision-making were guided by clinical and paraclinical judgment rather than standardized protocols, raising the potential for selection bias. In addition, PCR-positive results were not routinely confirmed with complementary diagnostic methods such as stool culture, antigen-based assays, and microscopy. Antimicrobial susceptibility testing was limited to culture-positive samples, as PCR-based detection of antimicrobial resistance (AMR) genes was not available in the participating hospital laboratories. The interpretation of co-infections also remains challenging, given the uncertain clinical relevance of multiple pathogen detection.

Another limitation is the absence of a cost-effectiveness analysis evaluating the integration of multiplex PCR into routine hospital practice. Although the upfront cost of multiplex assays is relatively high, potentially restricting adoption in resource-limited settings, these tests provide important clinical advantages, including a reduced need for ancillary investigations, improved antimicrobial stewardship through pathogen-directed therapy, and the facilitation of earlier therapeutic interventions. These benefits may ultimately translate into downstream cost savings and improved patient outcomes.

## 5. Conclusions

This retrospective study highlights important differences between traditional and PCR-based diagnostic strategies in the evaluation of hospitalized patients with suspected infectious diarrhea. Our findings demonstrate the superior diagnostic yield of multiplex PCR testing and emphasize its value in supporting antimicrobial stewardship. Future prospective studies are needed to assess cost-effectiveness, long-term clinical outcomes, and the significance of pathogens, particularly co-infections, detected by multiplex panels. Special consideration should be given to high-risk populations, including immunocompromised patients and those with complex comorbidities, in whom rapid and accurate pathogen identification is likely to provide the greatest clinical benefit.

## Figures and Tables

**Table 1:** Baseline characteristics.

Variable	Traditional Group (Hospital 1) (146)	Multiplex PCR Testing Group (Hospital 2) (121)	*p* Value
Age (median-IQR)	60 (36–73)	65 (53–75)	0.02
Min–max	18–96	19–95	
Sex, nr (%)			0.537
Male	69 (48.3%)	63 (52.1%)	
Female	73 (51.7%)	58 (47.9%)	
Length of hospital stay (median-IQR)	6 (4–11)	10 (6–15)	<0.0001
Min–max	1–45	3–39	
Charlson Comorbidity Index (median-IQR)	2 (0–5)	5 (2–7)	<0.0001
Min–max	0–10	0–12	
Comorbidities			
Cardiovascular disease	49 (35.8%)	59 (48.8%)	0.035
Liver cirrhosis	13 (9.4%)	25 (20.7%)	0.010
Respiratory disease	21 (15.2%)	19 (15.7%)	0.914
Renal disease	13 (9.4%)	31 (25.6%)	0.001
Diabetes	25 (18.0%)	21 (17.4%)	0.894
Inflammatory bowel disease	6 (4.3%)	12 (9.9%)	0.076
Cancer	23 (16.5%)	24 (19.8%)	0.492
Immunosuppressive medication	16 (11.5%)	42 (34.7%)	0.000
Recent surgery	11 (7.7%)	22 (18.2%)	0.008
Recent antibiotics	23 (16.9%)	14 (11.6%)	0.223
Symptoms			
Loose stools	71 (50%)	65 (53.7%)	0.547
Watery diarrhea	83 (60.6%)	44 (36.4%)	0.000
Bloody diarrhea	11 (8.0%)	34 (28.1%)	0.000
Abdominal pain	70 (51.1%)	93 (76.9%)	0.000
Dehydration	94 (68.6%)	62 (51.2%)	0.004

**Table 2:** Paraclinical investigations.

Variables	Traditional Group—Hospital 1 (146)	Multiplex PCR Testing Group—Hospital 2 (121)	*p* Value
WBC (median-IQR) (/µL)	8380 (6410–13,270)	11,000 (6725–14,460)	<0.0001
Hgb (g/dL)	12.85 (11.08–14.23)	11.2 (9.9–12)	<0.0001
CRP (mg/L)	4.35 (0.81–9.17)	109 (42–194)	<0.0001
Procalcitonin	0.5 (0–10)	0.5 (0.1–2.14)	0.19
Abdominal US	30 (21.58%)	30 (24.8%)	0.539
Abdominal CT scan	32 (25.36%)	41 (33.8%)	0.159
Endoscopy	9 (6.52%)	8 (6.61%)	0.964
Number of imagistic tests/patients (median-IQR)	0 (0–1)	0 (0–1)	0.068
Min–max	0–3	0–3	
Number of stool tests/patient (median-IQR)	2 (2–3)	1 (1–1)	<0.0001
Min–max	0–5	1–2	

**Table 3:** Diagnostic yield.

Pathogen Detected	Multiplex PCR Testing Group—Hospital 2 (*n = *121)	Traditional Group—Hospital 1 (*n = *146)	*p*
Any pathogen detected	94 (77.68%)	60 (41.09%)	0.0001
*Campylobacter*	56 (46.28%)	0	-
Single	24 (19.83%)		
Double	22 (18.18%)		
Triple	10 (8.26%)		
*C. difficile*	19 (15.70%)	22 (15.06%)	0.531
*Salmonella*	5 (4.1%)	33 (22.6%)	0.00001
Rotavirus	7 (5.78%)	3 (2.1%)	0.110
Single	4		
Double	1		
Triple	2		
Adenovirus	0	1 (0.68%)	-
*Aeromonas* spp.	12 (9.91%)	0	
Single	1 (0.82%)		
Double	7 (5.78%)		
Triple	4 (3.30%)		
*Blastocystis hominis*	18 (14.87%)	0	
Single	4 (3.30%)		
Double	9 (7.43%)		
Triple	5 (4.13%)		
*EPEC*	13 (10.7%)	0	-
Single	1 (0.82%)		
Double	9 (7.43%)		
Triple	3 (2.47%)		
*EHEC*	1	0	-
*STEC*	2 (1.65%)	0	-
*Shigella*	1 (0.82%)	0	-
Norovirus	3 (2.47%)	0	-
Sapovirus	1 (0.82%)	0	
*Yersinia*	1 (0.82%)	0	
*Cryptosporidium*	3 (2.47%)	0	
Giardia lamblia	3 (2.47%)	0	
*Dientamoeba fragilis*	3 (2.47%)	0	
*Strongyloides*	0	1 (0.68%)	
Mixed infections (≥2 pathogens)	42 (34.71%)	0	
Double pathogens	31 (25.61%)		
Triple pathogens	11 (9.09%)		

**Table 4:** Predictors of stool test positivity.

		Multiplex PCR Testing Positivity (Hospital 2)	
Predictor	OR	95% CI	*p* Value
Symptoms			
Watery diarrhea	0.18	0.02–1.52	0.114
Bloody diarrhea	16.5	1.81–150.26	0.013
Loose stool	1.15	0.21–6.11	0.874
Dehydration	7.05	1.4–35.45	0.018
CCI	1.04	0.87–1.24	0.668
Immunosuppression	0.54	0.17–1.76	0.31
CRP > 10 (mg/L)	4.52	0.34–60.83	0.256
WBC > 10,000/µL	0.48	0.16–1.44	0.19
		Traditional stool test positivity (Hospital 1)	
Predictor	OR	95% CI	*p* value
Symptoms			
Watery diarrhea	0.27	0.09–0.85	0.025
Bloody diarrhea	1.46	0.29–7.35	0.649
Loose stool	2.57	1.01–6.55	0.048
Dehydration	2.86	0.94–8.73	0.065
CCI	0.88	0.76–1.02	0.088
WBC > 10,000/µL	0.67	0.29–1.59	0.367
		Detection of mixed infections	
Predictor	OR	95% CI	*p* value
Symptoms			
Bloody diarrhea	0.89	0.34–2.31	0.806
Dehydration	0.79	0.31–2	0.623
ICU admission	2.01	0.68–5.95	0.205
WBC > 10,000/µL	1.5	0.62–3.64	0.371
CRP > 10 (mg/L)	1	0.99–1	0.063
Procalcitonin	1	1–1	0.465
CCI	1.05	0.91–1.21	0.542
Immunosuppression	1.28	0.52–3.14	0.596

**Table 5:** Empiric antibiotic use.

		Empiric Antibiotics Initiated Prior to Result	
Predictor	OR	95% CI	*p* Value
Symptoms			
Watery diarrhea	1.15	0.56–2.39	0.75
Bloody diarrhea	1.75	0.78–3.93	0.172
Abdominal pain	0.47	0.25–0.86	0.015
Dehydration	0.9	0.42–1.93	0.792
CCI	1.19	1.08–1.31	0.001
Comorbidities			
Cardiovascular	0.89	0.46–1.74	0.739
Liver cirrhosis	0.89	0.37–2.17	0.798
Respiratory	0.9	0.4–2	0.794
Renal	0.31	0.13–0.75	0.01
Diabetes	1.05	0.46–2.38	0.907
Cancer	1.9	0.73–4.99	0.191
Immunosuppressive therapy	1.12	0.45–2.75	0.808
Immunosuppressive status	1.09	0.64–1.84	0.756
WBC > 10,000/µL	2.18	1.33–3.6	0.002

**Table 6:** Inappropriate antibiotic use.

		Inappropriate Antibiotic Use	
Predictor	OR	95% CI	*p* Value
Symptoms			
Watery diarrhea	0.82	0.4–1.69	0.588
Bloody diarrhea	1.23	0.58–2.6	0.594
Abdominal pain	0.68	0.37–1.25	0.213
Dehydration	1.43	0.67–3.06	0.351
CCI	1.12	1.02–1.22	0.019
Type of stool test (PCR vs. traditional)	0.296	0.16–0.53	<0.001
Comorbidities			
Cardiovascular	1.47	0.75–2.91	0.263
Liver cirrhosis	1.1	0.46–2.65	0.831
Respiratory	1.4	0.63–3.13	0.409
Renal	0.36	0.15–0.84	0.018
Diabetes	1.31	0.58–2.96	0.52
Cancer	0.71	0.29–1.72	0.443
Immunosuppressive therapy	0.69	0.29–1.61	0.389
ICU admission	2.48	0.99–6.17	0.051
WBC > 10,000/µL	1.46	0.85–2.51	0.17

**Table 7:** Antibiotic (ATB) stewardship comparative analysis.

Variable	Traditional Stool Tests (Hospital 1) (146)	Multiplex PCR Testing Group (Hospital 2) (121)	*p* Value
Empiric ATB initiated prior to result	53 (36.3%)	84 (70.0%)	0.000
Empiric ATB continued after result	10 (6.84%)	0	
Change in ATB therapy after result	40 (27.39%)	51 (42.14%)	0.011
ATB discontinued after result	3 (3.70%)	15 (12.39%)	0.033
ATB added after result	31 (21.23%)	19 (15.70%)	0.248
Duration of ATB after result-median (IQR)	7 (5–10)	5 (0–7)	<0.0001
Min–max	0–12	0–10	

**Table 8:** Predictors of ICU admission and length of hospital stay.

		ICU Admission	
Predictor	OR	95% CI	*p* Value
Hospitalization days	1.08	1.02–1.14	0.006
Symptoms			
Watery diarrhea	0.83	0.27–2.58	0.75
Bloody diarrhea	2.04	0.72–5.79	0.18
Abdominal pain	0.79	0.3–2.1	0.641
Dehydration	1.42	0.45–4.49	0.553
CCI	1.26	1.1–1.43	0.001
Comorbidities			
Cardiovascular	0.42	0.16–1.12	0.83
Liver cirrhosis	6.69	2.52–17.77	<0.001
Respiratory	3.07	1.09–8.67	0.034
Renal	0.466	0.21–2.03	0.466
Diabetes	0.31	0.08–1.12	0.073
Cancer	0.92	0.24–3.47	0.904
Immunosuppressive therapy	2.34	0.75–7.32	0.143
Recent ATB	1.72	0.54–5.42	0.356
WBC > 10,000/µL	1.66	0.81–3.4	0.162
		Length of hospital stay	
Predictor	B	95% CI	*p* value
Positive stool test results	2.78	1.16–4.39	0.001
ICU admission	5.23	2.73–7.74	<0.001
Inappropriate ATB use	−1.01	−3.54–1.51	0.427
Symptoms			
Watery diarrhea	−0.22	−2.62–2.18	0.856
Bloody diarrhea	1.20	−1.37–3.78	0.355
Dehydration	1.59	−0.93–4.10	0.213
CCI	0.56	0.10–1.02	0.017
Comorbidities			
Cardiovascular	0.21	−1.92–2.34	0.846
Liver disease	2.44	−0.08–4.96	0.057
Respiratory	2.38	0.01–4.74	0.048
Renal	−4.15	−6.68–1.62	0.001
Diabetes	−2.92	−5.22–0.62	0.013
Cancer	−0.24	−3–2.53	0.866
WBC > 10,000/µL	2.67	0.84–4.50	0.004

## Data Availability

Data are available based on request from the corresponding author due to privacy and ethical restrictions from our institution.

## Abbreviations

The following abbreviations are used in this manuscript:

PCR	polymerase chain reaction
AMR	antimicrobial resistance
ICU	intensive care unit
LOS	hospital length of stay
AST	antimicrobial susceptibility testing
*C. difficile*	*Clostridioides difficile*
EHEC	Enterohemorrhagic *Escherichia coli*
EPEC	Enteropathogenic *Escherichia coli*
EIEC	Enteroinvasive *Escherichia coli*
STEC	Shigatoxigenic *Escherichia coli*
CCI	Charlson Comorbidity Index
SD	standard deviation
IQR	interquartile range
WBC	white blood cells
Hgb	hemoglobin
CRP	C reactive protein
US	ultrasound
CT	computed tomography
